# National survey on the presence of multidisciplinary meetings for interstitial lung diseases (ILD-MDM) in hospitals in Germany

**DOI:** 10.1007/s00393-025-01661-9

**Published:** 2025-09-06

**Authors:** Claus-Juergen Bauer, Dirk Skowasch, Michael Kreuter, Okka W. Hamer, Jürgen Behr, Sven Gläser, Claus Peter Heussel, Daniel Kütting, Andreas Krause, Gabriela Leuschner, Philipp Markart, Simon Michael Petzinna, Markus Polke, Valentin Sebastian Schäfer

**Affiliations:** 1 Clinic of Internal Medicine III, Department of Oncology, Hematology, Cell and Immunotherapies, Clinical Immunology and Rheumatology, University Hospital Bonn, Venusberg Campus 1, 53127 Bonn, Germany; 2 Medical Clinic and Polyclinic II, Section of Pulmonology, University Hospital Bonn, Bonn, Germany; 3 Lung Centre Mainz, Clinic for Pulmonology, Ventilation and Sleep Medicine, Marienhaus Clinic Mainz and Clinic for Pulmonology, University Medical Centre Mainz, Mainz, Germany; 4 Institute for Radiology, University Hospital Regensburg, Regensburg, Germany; 5 Medical Clinic and Polyclinic V, LMU University Hospital Munich, Munich, Germany; 6 German Centre for Lung Research, Giessen, Germany; 7 Vivantes Hospital Spandau and Neukoelln, Berlin, Germany; 8 Clinic for Diagnostic and Interventional Radiology, University Hospital Heidelberg, Heidelberg, Germany; 9 Translational Lung Research Center (TLRC), German Centre for Lung Research (DZL), Heidelberg, Germany; 10 Diagnostic and Interventional Radiology with Nuclear Medicine, Thorax Clinic, University Hospital Heidelberg, Heidelberg, Germany; 11 Clinic for Diagnostic and Interventional Radiology, University Hospital Bonn, Bonn, Germany; 12 Department of Rheumatology, Clinical Immunology and Osteology, Immanuel Hospital Berlin, Berlin, Germany; 13 Medical Clinic 5 (Pulmonology), Fulda Hospital, Campus Fulda, University Medicine Marburg, Fulda, Germany; 14 University Hospital Giessen and Marburg, Giessen, Germany; 15 Centre for Interstitial and Rare Lung Diseases, Pulmonology and Ventilation Medicine, Thorax Clinic, University Hospital Heidelberg, Heidelberg, Germany

**Keywords:** ILD-MDM, Lung diseases, interstitial, Interdisciplinary case conference, Rheumatology, Pulmonology, Radiology, ILD-Board, Interstitielle Lungenerkrankungen, Interdisziplinäre Fallkonferenz, Rheumatologie, Pneumologie, Radiologie

## Abstract

**Background:**

Interstitial lung diseases (ILD) represent an interdisciplinary clinical challenge and are not uncommonly associated with rheumatological diseases. Interstitial lung disease multidisciplinary meetings (ILD-MDM) provide a structured platform for interdisciplinary case discussions and decision making. Despite their great importance in patient care, data on the prevalence, structure and function of ILD-MDM in Germany are lacking.

**Objective:**

The aim of the study was to assess the current status of ILD-MDM in German hospitals to gain insights into their composition, processes and potential for optimization.

**Material and methods:**

A web-based survey was conducted via SurveyMonkey under the auspices of the German Society for Rheumatology and Clinical Immunology (DGRh) and in collaboration with the German Respiratory Society (DGP) and the German Radiological Society (DRG). A standardized questionnaire captured information on the participating specialist disciplines, organizational structures as well as the content and challenges of local ILD-MDM. The analysis was conducted descriptively.

**Results:**

A total of 125 physicians from 15 federal states in Germany participated. Pulmonologists (93.6%), radiologists (86.4%), rheumatologists (59.2%) and pathologists (57.6%) are the most commonly represented members of ILD-MDM. The majority of ILD-MDMs are conducted either in person (50%) or in a hybrid format (31.5%) and are held on a weekly basis (41.1%). Of all patient cases discussed, two thirds receive a definitive diagnosis and treatment recommendation.

**Conclusion:**

The findings reveal a high acceptance and prevalence of ILD-MDM in Germany but also highlight potential areas for improvement, particularly regarding interdisciplinary participation, technical infrastructure and standardization.

**Supplementary Information:**

The online version of this article (10.1007/s00393-025-01661-9) contains supplementary material.

## Abstract (de)

**Hintergrund:**

Interstitielle Lungenerkrankungen (ILD) stellen eine interdisziplinäre klinische Herausforderung dar und sind nicht selten mit rheumatologischen Erkrankungen assoziiert. ILD-Boards bieten eine strukturierte Plattform zur interdisziplinären Fallbesprechung und Entscheidungsfindung. Trotz ihrer hohen Bedeutung in der Patientenversorgung fehlt es an Daten zur Verbreitung, Struktur und Arbeitsweise von ILD-Boards in Deutschland.

**Ziel der Arbeit:**

Ziel der Studie war die Erfassung des Status quo von ILD-Boards an deutschen Kliniken sowie deren Zusammensetzung, Abläufe und Optimierungspotenziale.

**Material und Methoden:**

Es erfolgte eine webbasierte Umfrage via SurveyMonkey unter der Schirmherrschaft der Deutschen Gesellschaft für Rheumatologie und Klinische Immunologie (DGRh) und in Zusammenarbeit mit der Deutschen Gesellschaft für Pneumologie und Beatmungsmedizin (DGP) sowie der Deutschen Röntgengesellschaft (DRG). Ein standardisierter Fragebogen erfasste Informationen zu beteiligten Fachdisziplinen, organisatorischen Strukturen sowie Inhalten und Herausforderungen der lokalen ILD-Boards. Die Auswertung erfolgte deskriptiv.

**Ergebnisse:**

An der Studie nahmen insgesamt 125 Ärzte aus 15 Bundesländern teil. Pneumologen (93,6 %), Radiologen (86,4 %), Rheumatologen (59,2 %) und Pathologen (57,6 %) bilden die häufigsten Mitglieder von ILD-Boards. Mehrheitlich finden die erfassten ILD-Boards in Präsenz (50 %) oder hybrid (31,5 %) und 1‑mal wöchentlich (41,1 %) statt. Zwei Drittel der besprochenen Patientenfälle erhalten eine definitive Diagnosestellung und Therapieempfehlung.

**Diskussion:**

Die Ergebnisse zeigen eine hohe Akzeptanz und Verbreitung von ILD-Boards in Deutschland, jedoch auch Optimierungspotenziale, insbesondere in Bezug auf die interdisziplinäre Beteiligung, technische Infrastruktur und Standardisierung.

## Background and research question

Interstitial lung diseases (ILD) represent a significant clinical challenge in radiological diagnostics as well as in pneumological and rheumatological patient care.

Various rheumatological diseases (including systemic sclerosis, rheumatoid arthritis, and myositis) can be associated with ILD and can thus have a negative impact on prognosis [1]. Moreover, ILD can precede a later rheumatological systemic disease in about 10% of cases [2]. Furthermore, ILDs can arise from other identifiable causes (e.g. granulomatous diseases such as sarcoidosis, triggered by inhaled noxae/allergens such as hypersensitivity pneumonitis, or drug-induced) or occur idiopathically, i.e. without a recognisable cause. Following the already observed 87% increase in ILD mortality-related loss of life years between 1990 and 2017, a further doubling is expected in the next two decades [3].

As first recommended in 2002 in a joint statement by the American Thoracic Society and European Respiratory Society, the complexity of ILD diagnosis and treatment requires close multidisciplinary collaboration between pulmonology, rheumatology, radiology, and pathology, as well as other necessary disciplines as needed [4, 5]. In this context, interstitial lung disease multidisciplinary meetings (ILD-MDM) have become a valuable tool and gold standard to ensure coordinated, interdisciplinary decision-making [6, 7] and to optimise patient care.

ILD-MDMs provide a structured platform for discussing clinical cases, presenting imaging, histopathological, clinical, and laboratory findings, facilitating interdisciplinary diagnostic decision-making, and developing personalized treatment plans. Despite the growing importance of such multidisciplinary meetings in clinical practice, little is known about their prevalence, structure, and working methods in Germany. In particular, national data on aspects such as the composition of ILD-MDMs, the frequency and format of their meetings, and the challenges they face are lacking. This knowledge gap makes it difficult to identify best practices and highlight areas for improvement.

The present research project therefore aims to systematically capture the current status of ILD-MDMs at German hospitals.

## Study design and methods

Under the auspices of the German Society for Rheumatology and Clinical Immunology (DGRh) and in collaboration with the German Respiratory Society (DGP) and the German Radiological Society (DRG), the content of this study was developed and discussed in two online meetings involving a pneumological and radiological expert board within the DGRh working group “Lung involvement in rheumatological diseases” (Chair: Univ.-Prof. Dr. med. MUDr. Valentin S. Schäfer, University Hospital Bonn). The result was a questionnaire consisting of 26 individual questions. The list of German-language questions is presented in the electronic supplementary material online. The present study was approved by the Ethics Committee at the Medical Faculty of Bonn (IRB No. 288/23).

The web-based survey was conducted using SurveyMonkey® (SurveyMonkey Inc., San Mateo, CA, USA). An invitation to all members of the specialist societies was sent via the respective email distribution lists and newsletters of the DGRh, DGP, and DRG. Participation was possible between 10 May 2024 and 31 December 2024. Duplicate entries were excluded based on address and postal code data. After completion of the data completeness check, the statistical analysis was performed using descriptive statistical methods via SurveyMonkey®’s own tools and Microsoft Excel (Version 2411; Microsoft Corp. Redmond, WA, USA).

## Results

### Demographics

**Fig. 1:**
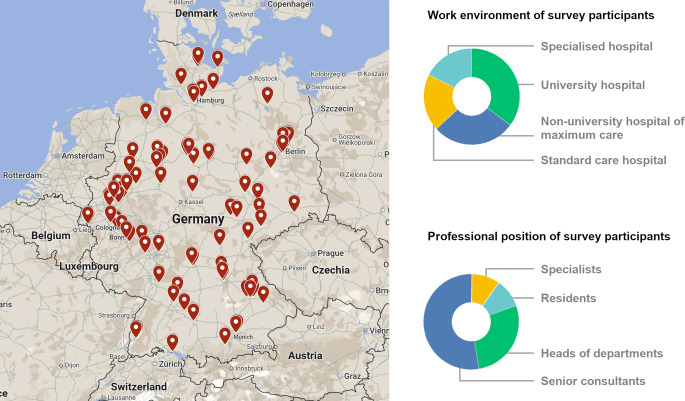
Survey participant demographics. Geographical location representation based on postcode query, type of institution, and professional demographics of the survey participants. Map material based on Google Maps (as of January 2025). © Google LLC, Mountain View, CA, USA. Graphic adaptation: Dr. Claus-Juergen Bauer, MD

A total of 125 participants completed the online survey. The completion rate was 100%. The average time taken was 6 min 8 s (standard deviation ± 37 min 59 s, or after removing 7 outliers with > 30 min processing time, which suggests a break during completion: ± 4 min 12 s). The participants consisted of 28% heads of departments, 52.8% senior consultants, 9.6% specialists, and 9.6% residents. Overall, rheumatologists, pulmonologists, and radiologists from 15 federal states participated, namely from North Rhine–Westphalia (24.0%), Bavaria (15.7%), Berlin (11.6%), Baden–Wuerttemberg (10.7%), Schleswig–Holstein (7.4%), Lower Saxony (6.6%), Rhineland–Palatinate (5.8%), Saxony (4.1%), Thuringia (4.1%), Hamburg (2.5%), Hesse, Bremen, Mecklenburg–Western Pomerania, and Saxony–Anhalt (each 1.7%), and Brandenburg (0.8%). Saarland was not represented. Four participants did not provide their geographical origin. The locations of the participants are mapped in Fig. 1. The majority of participants (35.2%) stated that they currently work at a university hospital. A further 28.0% work at a nonuniversity hospital of maximum care, 19.2% at a standard care hospital, and 17.6% at a specialised hospital. In further specification of the “employment at a specialised hospital” response, 17 participants stated that they work at a pneumologically specialised clinic, 2 participants at a rheumatologically specialised clinic, 1 participant at a rheumatologically and pneumologically specialised clinic, and 2 participants at a “specialised practice” without further details.

### Professional composition of the local ILD-MDM

**Fig. 2:**
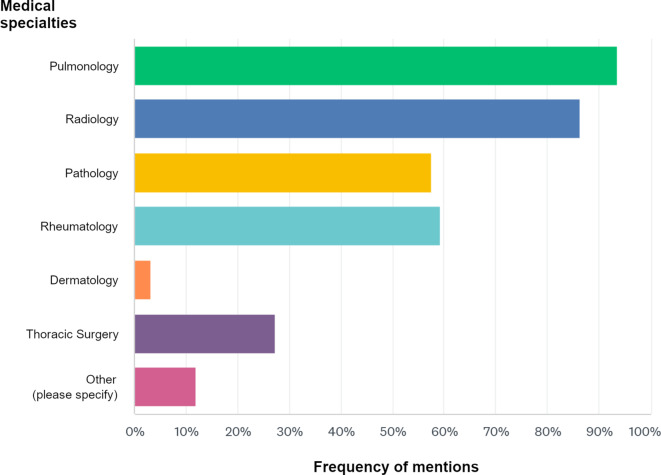
Professional composition of the interstitial lung disease multidisciplinary meetings (ILD-MDM). Frequency of mention of various disciplines as represented participants in the local ILD multidisciplinary meetings. Under “Other”, 3 respondents reported the participation of physicians in occupational medicine (2.4%), 2 indicated the regular participation of cardiology (1.6%), 2 noted the presence of nuclear medicine (1.6%), and 1 respondent reported the regular representation of transplant medicine (0.8%)

The most frequently represented disciplines in the local ILD-MDM were pulmonology (93.6%), radiology (86.4%), rheumatology (59.2%), and pathology (57.6%). Further response frequencies can be found in Fig. 2. Under “other”, 3 participants mentioned the presence of occupational medicine (2.4%), 2 participants mentioned the regular presence of cardiology (1.6%), 2 participants mentioned the presence of nuclear medicine (1.6%), and 1 participant mentioned the regular representation of transplantation medicine (0.8%). Additionally, 7 participants reported that individual disciplines are consulted as needed (particular specification: pathology *n* = 1, rheumatology *n* = 2, thoracic surgery *n* = 1).

Among all survey participants, 74.2% also stated that a minimum presence of disciplines is required for the ILD-MDM to take place at their own hospital. In 29 of 92 cases (31.5%), this was a combination of pulmonology and radiology, in a further 26 of 92 cases (28.2%) with the inclusion of rheumatology, and in a further 18 of 92 cases (19.6%) pulmonology and radiology with the inclusion of pathology. Seven of 92 participants (7.6%) stated that the minimum presence of the combination of pulmonology, radiology, rheumatology, and pathology is required. In 5 of 92 cases (5.4%), the minimum composition consisted of pulmonology, radiology, pathology, and thoracic surgery, in one further case (1.1%) with the additional presence of allergology, and in another case (1.1%) with the additional presence of nuclear medicine and rheumatology. Excluding the pathology department, the required minimum presence of pulmonology, radiology and thoracic surgery or of pulmonology, radiology, thoracic surgery and rheumatology was stated in 2 out of 92 cases (2.2%), respectively. In one case, minimum attendance was specified without radiology, solely by pneumology and rheumatology. In other words, among all ILD-MDMs that have defined a minimum participation of certain disciplines, individual disciplines are deemed mandatory in the following frequency: pulmonology (100%), radiology (98.9%), rheumatology (40.2%), pathology (34.8%), thoracic surgery (12.0%), nuclear medicine (1.1%), allergology (1.1%).

Among all 125 survey participants, 37 additionally stated that the pulmonology department in their own ILD-MDM must also be represented by at least 2 individuals. In one case, it was even stated that both pulmonology and rheumatology must be represented by at least 2 individuals.

### Spatial and temporal conception of ILD-MDMs

The ILD-MDMs surveyed nationwide in Germany are mostly conducted exclusively in person (50%) or in a hybrid format—i.e. both in person and virtually—(31.45%). In 18.55% of cases, the implementation is exclusively virtual. The most common meeting frequency is weekly (41.1%), followed by once a month (25.0%) or twice a month (17.7%). Four times, a twice-weekly ILD multidisciplinary meeting was reported (3.2%), and in one case (0.8%), the ILD-MDM even takes place four times a week. In 10.5% of the surveyed ILD-MDMs, the implementation takes place irregularly, depending on the number of cases.

The duration of an average ILD multidisciplinary meeting is 30–60 min at most locations (50.0%), 60–90 min (24.2%), or 15–30 min (18.6%), less often over 90 min (4.0%) or below 15 min (3.2%). The most frequently stated average number of patient cases discussed per ILD-MDM is “5–10 cases” (37.1%), “1–5 cases” (30.7%), or “10–15 cases” (23.4%); a higher number of cases is significantly less common (“15–20 cases”: 5.7%; “20–30 cases”: 2.4%; “> 30 cases”: 0.8%). Table 1 provides an overview of the characteristics of local ILD-MDMs in Germany in comparison to published data from European and global comparisons.

**Table 1:** Interstitial lung disease multidisciplinary meeting (ILD-MDM) characteristics in international comparison

ILD-MDM characteristic	Germany (%)	Europe (Source: [8]) (%)	Global (Source: [8]) (%)
Pulmonology presence	93.6	n. a.	99.7
Radiology presence	86.4	n. a.	91.4
Rheumatology presence	59.2	n. a.	37.1
Pathology presence	57.6	n. a.	66.3
Meeting at least every 2 weeks	62.8	60.8	66.9
Meeting at least weekly	45.1	n. a.	34.9
Conducted exclusively in person	50.0	81.1	80.0
Meeting duration: 31–60 min	50.0	48.6	50.9
Discussion of 1–5 patient cases per session	30.7	n. a.	61.0
Discussion of 5–10 patient cases per session	37.1	n. a.	29.0
Discussion of 10–15 patient cases per session	23.4	n. a.	8.0
Discussion of 15–20 patient cases per session	5.7	n. a.	2.0

This table compares the frequency of various ILD multidisciplinary meeting characteristics between ILD-MDMs in Germany, across Europe, and globally

*n.* *a.* not available

### Patient registration and access to ILD-MDMs

In 84.7% of all recorded ILD-MDMs, external physicians, including private practitioners and hospitals, have the option to register patients for discussion. On average, 14.7% (± 14.6) of the cases presented at ILD-MDMs originate from external referrals.

In a majority of sites (80.5%) patient registrations for the ILD-MDM are conducted using a standardized form. The most commonly requested patient-related data includes smoking status (90.3%), occupational history, autoimmune diagnostics, pathology reports, findings from the most recent computed tomography (CT) scan and CT pattern (each 87.6%), as well as medication history (86.7%), bronchoalveolar lavage findings (85.8%), body plethysmography results (84.1%), and symptom duration (81.4%). Less frequently required are specific immunoglobulin G (IgG) antibodies against antigens associated with hypersensitivity pneumonitis (72.6%), physical examination findings (61.1%), or C‑reactive protein (CRP) levels (42.5%).

### Process and structure of German ILD-MDMs

In only 0.8% of recorded ILD-MDMs, radiological findings are evaluated solely by reading out the written radiology report. In contrast, 99.2% of ILD-MDMs visually present radiological findings and engage in multidisciplinary discussions regarding ILD patterns. Histopathological findings are reviewed exclusively through written reports in 56.5% of cases, whereas 43.5% of local ILD-MDMs also include visual demonstrations and discussions of histopathological findings.

The vast majority of survey participants (97.6%) indicated that their ILD-MDM provides both a specific diagnostic classification and therapeutic recommendations. Additionally, 98.4% of respondents reported that their ILD-MDM includes recommendations for further diagnostics. Regarding this, 91.9% of participants stated that they and their multidisciplinary team typically follow the “S1 guideline on interdisciplinary diagnosis of interstitial lung diseases in adults” [9] when diagnosing ILDs. According to respondents, 32.6% (± 21.6%) of cases discussed at their ILD-MDM conclude with a recommendation for further diagnostics (e.g. cryobiopsy), while 30.5% (± 22.1%) result in a recommendation for follow-up CT imaging. Additionally, in 27.5% (± 15.9%) of cases, a referral for rheumatological assessment is suggested. A confirmed diagnosis is established in 62.0% (± 20.1%) of case discussions, and 69.3% (± 22.7%) of cases receive a therapeutic recommendation.

Following the discussion of a patient case, a standardized outcome report is generated in approximately two-thirds (66.1%) of the documented ILD-MDMs. However, a final decision on a patient case is not always possible at the time of the ILD multidisciplinary meeting. On average, 12.1% (± 12.1%) of cases require postponement due to missing findings. To address such cases, follow-up patient reviews are conducted in 91.9% of national ILD-MDMs, for example, after a cryobiopsy was performed or in cases of clinical deterioration.

### Approaches to improving future ILD-MDMs

The online survey concluded with an open-text question: “How could future ILD-MDMs be further improved?” A total of 78 out of 125 respondents (62.4%) provided written feedback. The most frequently mentioned suggestions and requests fell into four overarching categories.

In 20 of 78 responses (25.6%), participants expressed a desire for the inclusion of additional medical specialties, with rheumatology (10/78; 12.8%) and pathology (10/78; 12.8%) being the most frequently requested disciplines.

The second most frequently mentioned suggestion was the enhancement or establishment of technical infrastructure for virtual ILD-MDM participation, advocated by 16 of 78 respondents (20.5%). A major concern in this regard was ensuring compliance with data protection regulations. The concept of a virtual or hybrid ILD-MDM was also repeatedly linked to the suggestion of opening participation to external physicians, such as rural healthcare practitioners or specialists outside the hospital setting (7/78; 9.0%).

The third most common request (12/78; 15.4%) was for increased standardization, particularly concerning the registration process and forms. One respondent suggested that “ILD-MDMs should only be permitted at certified centres”.

The fourth most frequently addressed category (7/78; 9.0%) related to economic aspects. The primary concern was improving the financial reimbursement of ILD-MDMs, while fewer responses mentioned the need for strengthened personnel and time resources for planning, case collection, and documentation of results.

## Discussion

This study provides a comprehensive national assessment of the structure, organization, content, and challenges of ILD-MDMs in Germany. As the first structured investigation of its kind in Germany, it offers valuable insights into the composition, workflow, and potential for further development of these key discussion platforms for the diagnosis and treatment of interstitial lung diseases (ILDs).

The near-universal presence of pulmonology and radiology in ILD-MDMs, as documented in our study, underscores their central role and aligns with global data reported by Richeldi et al. in 2019 [8]. Rheumatology and pathology are integral components of nearly two-thirds of ILD-MDMs in Germany. Notably, the presence of rheumatology in German ILD-MDMs exceeds international figures (survey results: 59.2% vs. global: 37.1% [8]). The above-mentioned disciplines fundamentally form the backbone of interdisciplinary diagnostics [4, 5]. However, the limited participation or availability of rheumatology and pathology in some regions—especially compared to pulmonology and radiology—represents a key area for improvement, as supported by the frequent requests from survey participants for stronger involvement of these specialties. The partial lack of pathology participation in ILD-MDMs may be linked to the absence of cryobiopsy procedures in certain locations, which results in a lack of histological findings as a basis for discussion. However, particularly centres with established ILD-MDMs—by virtue of their demonstrated expertise in the management of patients with ILD—should be supposed to offer access to cryobiopsy, as being currently considered the first-line biopsy technique in cases of suspected fibrosing ILD [9, 10], at least through cooperative arrangements. Furthermore, the German S1 guideline on “Interdisciplinary diagnosis of interstitial lung diseases in adults” emphasizes: “Mandatory participants are experienced professionals in the field of ILDs from pneumology, (thoracic) radiology, and (thoracic) pathology (if histopathology is available)” [9]. Particularly in cases where a definitive diagnosis cannot be made based on clinical, anamnestic, and radiological data alone, histopathological evaluation often plays a crucial role and can significantly alter the assessment made by clinicians and radiologists [6]. Previous studies have shown that enhanced multidisciplinary collaboration can lead to better diagnostic and therapeutic decisions [6, 11]. Given that ILD can be the first manifestation of rheumatologic diseases in up to 10% of cases [2], closer integration of rheumatology appears to be particularly crucial.

Additional characteristics of ILD-MDMs in Germany, particularly their timing and frequency, align closely with European comparison data from 2019. For instance, the proportion of ILD-MDMs meeting at least every 2 weeks is similar (Germany: 62.8% vs. Europe: 60.8% [8]) , as is the typical meeting duration (31–60 min: Germany: 50.0% vs. Europe: 48.6% [8]). However, the proportion of ILD-MDMs that only allow in-person participation has declined significantly from 81.1% (reported in 2019) to 50.0% in Germany, likely influenced by the COVID-19 pandemic. This shift has particularly benefited patients and healthcare providers in rural areas by improving accessibility through enhanced digitalization and virtualization. Nevertheless, many survey respondents still emphasized the need for technical solutions that ensure data protection compliance to facilitate widespread virtual or hybrid participation in ILD-MDMs. The effectiveness of virtual participation in improving accessibility and multidisciplinary collaboration has already been demonstrated in virtual tumour boards [12].

Investments in digital infrastructure and virtual transmission channels, as well as opening ILD-MDMs to external participants, are measures that must also be considered from an economic perspective, as highlighted by survey participants. However, German lung centres certified by the G‑BA could serve as pioneers in this regard, as their ILD-MDMs often emphasize external participation as a key feature. In particular, the financial sustainability of these time- and resource-intensive meetings requires greater attention in health economics. Previous studies have demonstrated that multidisciplinary case discussions can be cost-effective in the long term by reducing misdiagnoses and improving treatment quality [11]. A key approach to reducing organizational workload, optimizing resource use, and ensuring quality control lies in standardizing the registration process and documentation. Survey participants frequently expressed the desire for greater standardization, especially through the use of uniform registration forms and ILD-MDM certification. Indeed, this demand for more clearly defined implementation guidelines aligns with the currently limited definitions of minimum standards and quality criteria for ILD-MDMs found in global scientific literature. While there is broad consensus among international guidelines regarding some overarching principles—such as the interdisciplinary composition of ILD-MDMs with a minimum professional presence of pulmonology, radiology, pathology, and, on a case-by-case basis, rheumatology —more specific minimum standards for ILD-MDMs are largely pending [13]. Increased standardization, particularly regarding patient case registration data, could significantly reduce the current average rate of 12% of patient cases whose discussion must be postponed due to missing findings. A significant step toward this goal has already been made with the S1 guideline “Interdisciplinary diagnostics of interstitial lung diseases in adulthood” [9], which provides recommendations for standardized ILD-MDM registration and protocol forms. The Working Group on Pulmonary Involvement in Rheumatic Diseases of the DGRh aims to address the challenges of adaptation and implementation in order to establish a standardized form and to validate its application. Greater standardization could also facilitate the future integration of artificial intelligence, for example, in the registration process and case preparation.

In conclusion, the high frequency of diagnosis and treatment recommendations resulting from ILD-MDMs underscores the effectiveness of multidisciplinary case conferences in clinical decision-making for interstitial lung diseases. This further reinforces the established role of ILD-MDMs as the “gold standard” in the optimized management of interstitial lung diseases [13, 14].

A key strength of this study lies in its comprehensive national data collection involving various professional societies. The high completion rate (100%) and broad regional representation enhance the generalizability of the findings.

However, the study has certain limitations. Data collection was conducted via an online survey, preventing objective validation of responses. Voluntary participation may have led to an overrepresentation of particularly engaged or well-established centres. Furthermore, the study relied on self-reported data from participants, which could introduce biases related to subjective perceptions.

Overall, this study highlights the importance of ILD-MDMs in Germany and provides the first comprehensive insights into their structure and workflow. The results emphasize the need to address existing challenges, such as integrating additional specialties, advancing digitalization, and standardizing processes, to enhance the effectiveness of these platforms and ensure an adequate standard of quality. Through stronger networking and continuous further development, ILD-MDMs could make an even greater contribution to improving the care of patients with interstitial lung diseases.

## Key takeaways for clinical practice

- Interstitial lung diseases multidisciplinary meetings (ILD-MDM) are an essential tool for the diagnosis and management of interstitial lung diseases and enable well-founded clinical decision-making.
- This study provides the first comprehensive insights into the structure, organisation, and challenges of ILD-MDMs in Germany, identifying key areas for improvement.
- Greater standardisation—particularly in patient registration—along with the expansion of hybrid and virtual formats, could enhance efficiency and accessibility.
- Stronger involvement of additional specialties, such as rheumatology and pathology, is crucial for improving diagnostic and therapeutic quality.
- The continuous evolution of ILD-MDMs through standardisation, digitalisation, and artificial intelligence holds significant potential for future advancements in patient care.

## Supplementary Information

The list of German-language questions

## Data Availability

Data will be made available on reasonable request.

## Declarations

The present study was approved by the Ethics Committee at the Medical Faculty of Bonn (IRB No. 288/23).

The supplement containing this article is not sponsored by industry.
